# Effect of exercise training on long-term potentiation and NMDA receptor channels in rats with cerebral infarction

**DOI:** 10.3892/etm.2013.1319

**Published:** 2013-09-27

**Authors:** QIAN YU, XIAOHONG LI, JING WANG, YUFENG LI

**Affiliations:** 1 Department of Rehabilitation, Sichuan Academy of Medical Sciences & Sichuan Provincial People’s Hospital, Chengdu, Sichuan 610072, P.R. China; 2 Department of Neurology, Affiliated Hospital of Luzhou Medical College, Luzhou, Sichuan 646000, P.R. China; 3 Department of Nutrition, Sichuan Academy of Medical Sciences & Sichuan Provincial People’s Hospital, Chengdu, Sichuan 610072, P.R. China

**Keywords:** cerebral infarction, exercise training, long-term potentiation, N-methyl-D aspartate receptor channel

## Abstract

The aim of the present study was to investigate the effects of exercise training on the characteristics of long-term potentiation (LTP) and N-methyl-D aspartate (NMDA) receptor channels in the hippocampal CA3 neurons of rats with cerebral infarction. Wistar rats were randomly allocated into the model without any training and rehabilitation with exercise training. A model of cerebral infarction was established by middle cerebral artery occlusion. Using chronically embedded electrodes combined with an electrophysiological method, the population spike (PS) amplitude and latency, as well as changes in the NMDA single channel current in the hippocampal neurons were determined prior to and following Y-maze discrimination learning 60 times in the two groups. The formation of learning-dependent LTP and synaptic efficacy in the hippocampal CA3 area after exercise training in the rehabilitation group was significantly faster compared with that in the model group without any training (P<0.05). The incubation period of the PS in the CA3 area of the rats in the rehabilitation group was significantly shorter compared with that in the model group. The PS amplitude in the rehabilitation group was significantly higher compared with that in the model group. Furthermore, the opening probability of the NMDA receptor channel in the rehabilitation group was significantly higher compared with that in the model group. In conclusion, exercise training improved the opening conductance level, time and probability of NMDA receptor channels and accelerated the formation of learning-dependent LTP in the contralateral hippocampal CA3 area.

## Introduction

The majority of patients with cerebral infarction have learning and memory dysfunction that seriously affects their quality of life. Exercise training may have a positive impact on many physiological systems, including the central nervous system. Exercise training has been shown to promote motor function and to improve learning and memory in rats with infarction (1). The mechanism of action is directly associated with activity-dependent synaptic plasticity and changes in gene expression (1,2). In one study, exercise training was reported to enhance the peak potential of long-term potentiation (LTP), the neural basis of learning and memory (2). LTP occurred as the synergistic result of neurotransmitter release in the presynaptic membrane and opening of the N-methyl-D aspartate (NMDA) receptor channel in the synaptic membrane.

Currently, rat or mouse hippocampal CA1 cells are widely used as research subjects in the basic study of LTP and NMDA receptor channels when investigating the learning and memory of experimental animals. However, nerve cells in the hippocampal CA3 area, i.e., associative memory in experimental animals, are rarely used as research subjects. One of the main functions of memory is to establish connections among associated experiences. The greater the amount of active association that takes place in thinking, the firmer the connection of experiences is likely to be. The effect of memory may be enhanced by the frequent formation and use of associations (3). Therefore, based on the above-mentioned concepts, the aim of the present study was to further investigate the impact of exercise training on the characteristics of LTP and NMDA receptor channels in the hippocampal CA3 neurons of rats with cerebral infarction, and to elucidate the neural mechanisms responsible for the improvements in learning and memory recovery following exercise training in rats with cerebral infarction.

## Materials and methods

### Animals

Healthy male Wistar rats (weight, 250±50 g; age, 8 weeks), obtained from the Experimental Animal Center of Luzhou Medical College (Luzhou, China), were randomly allocated into two groups: the model (n=8, free activity after cerebral infarction) and the rehabilitation group (n=8, exercise training after cerebral infarction). The animal protocol was reviewed and approved by the Institutional Animal Care and Use Committee (IACUC) of the Sichuan Academy of Medical Sciences & Sichuan Provincial People’s Hospital (Chengdu, China).

### Electrode implantation and model preparation

The rats were anesthetized with an intraperitoneal injection (3 ml/kg) of a mixture of 25% urethane (Shanghai Hengyuan Biotechnology Co., Ltd., Shanghai, China) and 1% chloral hydrate (Shanghai Sunshine Biotechnology Co., Ltd., Shanghai, China) dissolved in water and fixed in a stereotaxic apparatus (RD1617-1SS; Shanghai Yishu Information Technology Co., Ltd., Shanghai, China). Electrodes were implanted under aseptic conditions. Briefly, a scalp incision was performed and the left top part of the cranium was exposed. The skull was drilled at locations in the hippocampus and entorhinal area to form a small hole in each for the feedthrough fiber perforant path (PP). The recording electrode, which comprised an insulated metal electrode (diameter, 0.2 mm; tip impedance, <5 kΩ), was implanted in the hippocampal CA3 pyramidal cell layer using a micro-electrode propeller (XX11-WK-III; Beijing Sunny Instruments Co. Ltd., Beijing, China). The recording electrode was calibrated by the rat brain in stereotaxic coordinates (4) and positioned [behind the bregma (Ap), 3.0–3.4 mm; left side of midline (L), 3.3–3.5 mm; depth (H), 3.8–4.2 mm]. The stimulating electrode was twisted together with two insulated Nichrome wires (diameter, 0.14 mm). The tips were exposed by 0.2–0.3 mm and the distance of the two tips from top to bottom was 0.5–0.6 mm. The feedthrough fiber (PP) was implanted in the entorhinal area and positioned at Ap, 6.8–7.0 mm; L, 4.4–4.5 mm; H, 5.5–6.2 mm. The reference electrode was fixed with a screw in the skull. As the electrodes were implanted, the recording and stimulating electrodes were moved up and down by the targeting parameters to find the site at which the largest group of population spikes (PS) could be recorded, before finally fixing with denture powder.

Rat models with ischemic infarction in the right middle cerebral artery were established according to the method of suture embolism described by Koizumi (5). Briefly, an incision was made in the right neck and the right common carotid artery, and the internal carotid artery and carotid artery were separated. A vascular clip was placed at the beginning of the pterygopalatine artery, the external carotid artery was ligated and a small opening of 0.2 mm in diameter was cut. The external carotid artery was ligated using a vascular clip that had been boiled with gentian violet in advance and dyed purple. A nylon fishing line, also dyed purple by boiling with gentian violet in advance, was coated uniformly with a layer of polyurethane varnish 4 mm away from the head end (<0.25 mm in diameter) in order to make it into a smooth taper. The suture embolism was inserted into the external carotid artery from its stump, through the carotid bifurcation, along the internal carotid artery and finally into the anterior cerebral artery in the skull. The insertion was stopped when a little resistance was felt and the insertion depth from the bifurcation was ~17–19 mm. Thus, the infarction model with a middle cerebral artery occlusion was prepared. The rat rectal temperature was maintained at 37°C using a thermostatic water bag during the surgical process. After regaining consciousness, the rat showed flexion and adduction of the left forelimb when suspended by the tail, ipsilateral Horner’s sign, circling to the left when crawling and falling to the left side. The rats that exhibited the above four signs were indicated to have undergone successful model preparation and were included in the study.

### Exercise training

Rats in the rehabilitation group had a break for 4 days and began to exercise training on the fifth day. The training methods used were the following: i) drum-type reticular training. The rats were put into a round reticular instrument (length, 100 cm; diameter, 60 cm). The instrument was rotated by hand to allow the rat to passively run along the cage to train the rat’s abilities to grip, rotate and walk. ii) Balance training. The rat was placed on a square wooden stick (length, 150 cm; width, 2 cm) 5 cm above the ground and was induced to walk by food. iii) Mesh barrier training. The rats were put in a mesh barrier (size of mesh, 1×1 cm; wire width, 50×40 cm). The height from the ground was 80 cm and sponge with a thickness of 12 cm covered the ground. When the mesh barrier was shifted from a horizontal to a vertical position step by step and maintained for 5 sec, whether the rat fell from the net was observed and the time that the rat held the net before falling from the net to the sponge was recorded. The longer the time, the better the muscular strength. Thus, grasping ability and muscle strength were trained. The time for each of the above training exercises was 30 min, 2 times per day. The training was carried out for 6 days of each week and lasted for 4 weeks.

### Behavioral tests and LTP recording in vivo

The Y-maze is a three-arm maze with a signal lamp on the top of each arm. If a lamp is turned on, the respective arm becomes the danger zone 6 sec later. The rat was stimulated by 36 V of AC power to run from the bright arm to the dark arm. One arm was randomly used as a safe area. The result was recorded as an error when the rat ran from one bright arm to another bright arm after the power was turned on, or recorded as correct when the rat ran to the dark arm. The rats were trained in continuous subsections within a day. Ten repetitions were considered as one section and the rats had a break of 2 min between two sections. The required training times were recorded for grasping the maze structure (success in nine of the 10 repetitions of the tests in succession). The fewer the number of training times required, the stronger the learning ability of the rat was. This was used as an indicator to judge the rat’s ability for learning and distinguishing.

Regarding LTP recording *in vivo*, prior to and 4 h after the training, learning and memorizing behavior tests, the rats were kept in a quiet state in a self-made cage. Stimulation was detected as a single square wave pulse (0.1 msec wave width) with fixed stimulus intensity acting on the PP fiber. The fixed intensity stimulation indicated that the model group was stimulated by stimulators and isolators (0.7 Hz, 0.1 msec wave width, single square wave pulse) before the Y-maze discrimination and learning tests, and the stimulus intensity was equivalent to 50% of the strength of the maximum amplitude of the peak potential causing the synaptic response. LTP was recorded in the CA3 pyramidal cell layer. The evoked potential was acquired and analyzed by a computer after amplifying with a WFI microelectrode amplifier, DSJ-F physiological amplifier and A/D card amplifier (Chengdu Technology & Market Co., Ltd., Chengdu, China). The PS was the vertical distance between the crest and the lowest point of the connected trough. The synaptic transmission was calculated from PS amplitude average differences, by dividing their corresponding averages of 4 h after and before the model group’s behavioral tests. Therefore, the LTP effect indicators were evaluated as the relative percentage of the PS amplitude. The amplitude amplified percentage was compared before and after the learning behavior, and the LTP was assessed by a t-test to determine whether there was a significant difference.

### Neuron NMDA receptor single channel

According to the reported methods for the acute isolation of neurons from the hippocampus of adult rats (6), the rats were anesthetized with ether, the brains were obtained by decapitation, and then the left hemisphere of the brain was immediately placed in 4°C Hank’s liquid without Ca or Mg, and ventilated with mixed gas (95% O_2_ + 5% CO_2_). After cooling equilibration, the brain portion was placed on filter paper dipped in Hank’s solution, and the hippocampus was separated. Five to eight pieces with a thickness of 0.4–0.5 mm were cut perpendicularly to the long axis of the hippocampus with a razor. The pieces were placed in artificial cerebrospinal fluid (ACF) to balance for 1 h. The constitution of the ACF was NaCl (126 mmol/l), KCl (5 mmol/l), NaHCO_3_ (25 mmol/l), NaH_2_PO_4_ (1.5 mmol/l), CaCl_2_ (24 mmol/l), MgSO_4_ (2 mmol/l) and glucose (GS; 10 mmol/l). The pH value was 7.4, the temperature was maintained at 32°C, and the gas mixture was continuously ventilated. The brain slices after cooling equilibration were digested with ACF containing 3 mg Pronase. After washing with ACF three times, the CA3 region was separated. Subsequently, a filiform sample of the CA3 region was isolated with a needle, transferred into 2 ml Dulbecco’s modified Eagle’s medium (DMEM) and continuously pipetted to scatter organizations and free cells with a polished Pasteur pipette tip (from coarse to fine with diameter from 500 to 150 μm) at room temperature. After a 2-min rest, the upper cell suspension was transferred onto the coverslip prepared in advance, coated with 0.1% poly-L-lysine, dried and then left to stand for 10 min. After the cells became adherent, the excess tissue debris and non-attached cells were rinsed away with DMEM solution prior to the addition of bath solution [composition (mmol/l): NaCl 140, CsCl 5, CaCl_2_ 1.8, GS 10, tetrodotoxin (TTX) 0.001 and HEPES 10, pH 7.2]. Neuron morphology was observed under an inverted microscope. The neuron cells with good adherence, a complete membrane, clear nucleus, a conical shape, strong three-dimensional geometry and neurites were chosen. A micro-propulsion drive electrode was used to approach the cells. A slight negative pressure was used at the moment when the micro-electrode tip made contact with the cells. Thus, a high pressure seal was formed instantly (impedance >10 GΩ)which constituted the cell-attached channel recording. The sugar-free electrode liquid included 20 μM NMDA, 1 μM glycine and other ingredients that were identical to those in the bath solution. The glass microelectrode for recording was drawn by a PP-83 microelectrode puller, and the glass blank was a GG17 hard blank developed by the Shanghai Brain Research Institute, Chinese Academy of Sciences (Shanghai, China). The first pull current was 13.0 mA, and the second pull current was 9.0 mA. The homemade polished electrode tip was ~1 μm in diameter, with an impedance of 8–15 MΩ and sealing resistance >10 GΩ. The current was enlarged by a CEZ-2200 diaphragm embedded amplifier (Nihon Kohden, Tokyo, Japan). The sampling process was continuously carried out by Fetchex program (Molecular Devices, Sunnyvale, CA, USA). The sampling frequency was 20 kHz, the low-pass filtering frequency was 1 kHz and the experimental temperature was 21–24°C.

Measurement was performed through the switching events of Fetchan procedures in pClamp 6.0 software (Molecular Devices). The measurement results were analyzed with a pSTAT program (Molecular Devices). Gaussian and exponential distribution histograms were used to fit the magnitude of the current channel and the channel switching time. Each fitting was compared by Marquardt-LSQ, Simplex-LSQ and Simple-MLE statistical methods. The experimental data were analyzed using single-factor analysis of variance, and the results were expressed as the mean ± standard deviation (SD). P<0.05 was considered to indicate a statistically significant result

## Results

### Comparison of Y-maze discrimination and PS increase in the hippocampal CA3 area after learning

The peak PS significantly increased (P<0.05) after the rats had repeated the learning and training exercise 60 times, indicating that the LTP effect in the exercise group was stronger compared with that of the model group. When the Y-maze discrimination learning of the exercise and model groups had reached the required standard, the difference between the PS amplitudes of the two groups was not significant (P>0.05), indicating that the same LTP was obtained after certain types of behavior (Table I).

### Comparison of the PS peak latency of the hippocampal CA3 area after Y-maze discrimination learning

The mean peak latency of the exercise group was significantly shorter compared with that of the model group after Y-maze discrimination learning (P<0.05). The PS value of the exercise group increased after balancing, gripping, rotating and walking training. This indicated that greater LTP was generated when the rats had undergone Y-maze learning and showed that the synaptic effect was enhanced (Table II). The result was consistent with behavior detection, indicating that training enhanced learning acquisition.

### Current and dynamic characteristics of the NMDA single channel of hippocampal neurons

The NMDA receptor channel of the exercise group had two conductance states (20 pS and 35 pS). The main channel conductance was usually 35 pS, and there was no long channel 35 pS. The characteristics of the recorded 20 pS and 35 pS conductance channels of the model group, compared with those of the exercise group were as follows: the conductance was mainly 20 pS and there was no 35 pS long channel; the short opening and long opening constants of the 20 pS conductance channel were significantly shorter compared with those of the exercise group (P<0.05); lower open probability; the short opening time constant and open probability of the 35 pS channel in the model group were also lower compared with those of the exercise group (Table III).

## Discussion

LTP is a manifestation of synaptic plasticity, which reflects information storage at the synaptic level. LTP is the objective indicator of the physical activity of neurons in processes relating to the memory. Previous studies have shown that the NMDA receptor plays an important role in learning or memory and the LTP process. The spatial learning and memory ability of rats was significantly decreased in the Morris water maze (7) and eight-arm radiation maze (8) when MK80, dextromethorphan and other antagonists were used to block NMDA receptors. Melik *et al*(9) showed that impairment of fear memory and the clear clue memory retrieval process occurred upon dysfunction of the NMDA receptor of the hippocampus in experimental animals, suggesting that the NMDA receptor plays an important role in the extraction of spatial and episodic memory.

The induction and maintenance of LTP requires the involvement of NMDA receptors. LTP is the result of synergistic action at the synaptic level, which depends on presynaptic neurotransmitter release and postsynaptic membrane NMDA receptor channel opening. When the receptor is blocked, LTP is not able to be induced (10). The NMDA receptor is also involved in the retention of LTP (11). The two major factors that induce LTP are the frequency and intensity of tetanic stimulation. A certain intensity of stimulation increases the amplitude of the single stimulation excitatory postsynaptic potential (EPSP), while a certain frequency of stimulation generates an additive effect on EPSP. This process results in a particular intensity of postsynaptic membrane depolarization, and the removal of the Mg^2+^, which can block Ca^2+^ influx in the NMDA receptor channel (12). Thus, the receptor channel opens when the neurotransmitter glutamate and NMDA receptor bind; Ca^2+^ influx occurs; calmodulin kinase, cAMP-dependent protein kinase and mitogen-activated protein kinase (MAPK) are activated, and these kinases activate cAMP response element binding protein (CREB), initiating a series of biochemical reactions, gene transcription and the induction and maintenance of LTP (13,14). Notably, gene expression and protein synthesis are not required for the formation of early LTP, while new protein synthesis is required for late LTP (LTP maintenance) and long-term memory formation. Gene expression is initiated by the binding of the NMDA receptor with target genes in the nucleus through the downstream signaling pathways of the receptor to induce LTP maintenance and memory (12). Murphy and Glanzman (15) confirmed the activation of NMDA receptors in LTP generated by establishing the gill withdrawal reflex of the California sea hare (*Aplysia californica)*. This study identified that spatial memory storage was usually generated by the activation of the NMDA receptor, and that the induction of LTP in *Aplysia californica* was inhibited by the ventricular injection of competitive and non-competitive NMDA receptor antagonists. Zhang *et al*(16) also demonstrated that NMDA receptor activation was required for the induction of LTP. In this study, the role of NMDA receptors in LTP was also confirmed by gene knockout. Therefore, both the induction and maintenance of LTP depend on presynaptic neurotransmitter release and postsynaptic NMDA receptor channel opening, which is the result of the synergistic action between the two processes at the synaptic level. Moreover, the postsynaptic NMDA receptor channel was shown to play a main role in LTP generation in the two processes.

The activation of NMDA receptors is required in enhancing synaptic plasticity, as well as in learning and memory processes by training (17). Dietrich *et al*(18) reported that the level of phosphorylation of NR1 and NR2B subunits of the rat cerebral cortex NMDA receptor was significantly upregulated and that the NMDA receptor was activated by self-running wheel activity for 1 month. The NMDA receptor channel open rate is also increased by training. Recently, additional studies have shown that brain-derived neurotrophic factor (BDNF) upregulates the function of the NMDA receptor, and that exercise increases the level of brain BDNF expression (13). Thus, the phosphorylation levels of the NR1 and NR2B subunits of the NMDA receptor are increased in the hippocampus and cerebral cortex (19), and the receptor ion channel opening and information transmission are increased (20). Molteni *et al*(21) investigated the influence of a self-running wheel exercise on gene expression in the rat hippocampus. The results obtained indicated that the expression levels of NR1, NR2A and NR2B mRNA in the rat hippocampus were significantly increased after 3 and 7 days of movement. Furthermore, the expression levels of the genes mentioned above in the exercise group continued to be higher compared with those of the control group after 28 days of exercise. Farmer *et al*(22) showed that dentate nuclear LTP is induced and that the expression levels of NR2B in the hippocampus are increased by self-running wheel exercise. Previous studies (17,23) suggest that the NMDA receptor easily accepts neurotransmission when there are high NR1 and NR2 expression levels in the brain, which are associated with enhanced learning and memory ability. Additional studies have confirmed that the NR1 and NR2B gene expression levels in the hippocampus, as well as the learning and memory abilities of experimental animals are differentially improved after 1 month of voluntary movement (24), suggesting that appropriate training improves the gene expression levels of the NR1 and NR2B subunits of the NMDA receptor and enhances the function of the NMDA receptor, which plays a catalytic role in learning and memory ability. Another study has shown that induction of NMDA receptor-dependent LTP is enhanced by increasing the NMDA receptor density (25).

The present study indicated that 20 pS conductance of the NMDA receptor channel in the model rats was the main mode, while 35 pS conductance of the NMDA receptor channel was significantly reduced. Apart from the difference in the number of two conductance channels opening, both the open duration and the open probability of the 35 pS conductance were decreased, and the short opening and long opening constants of 20 pS conductance in the model group were significantly shorter compared with those of the exercise group. The results of the LTP measurement also showed that the PS peak amplitude in the two groups of rats increased after Y-maze learning 60 times, indicating that learning-induced LTP occurred in the two groups of rats. The percentage of PS increase was greater and the peak latency was shorter in the exercise group than in the model group, indicating a significant difference. However, the increase of PS in the two groups was comparable when the level of correct discrimination in the Y-maze test reached >90%. Possible reasons may be the essence of rehabilitation training, such as balancing, gripping and rotating, which were relearned by the hemiplegic rats by movement. Moreover, learning-induced LTP occurred in the rehabilitation group due to these training procedures. Based on these results, LTP may be increased following Y-maze learning, and the development of the PS peak soon reached saturation.

Therefore, in the rat model of cerebral infarction, the structural parameters of the contralateral brain synaptic interface were altered and the LTP peak latency was shortened following training. These alterations resulted in the conductance level, opening times and open probability of the contralateral hippocampal CA3 NMDA receptor channel and accelerative development of learning-induced LTP in the hippocampal, which improved learning efficiency and the restoration of learning and memory.

Although important information concerning LTP has been obtained, further studies are required. In a previous study (26), similar to the current study, which focus on the impact of exercise training on the hippocampi of rodents have been conducted. However, the effects of exercise training on LTP in the brain and other organs of primates and in other brain areas have not yet been fully investigated. The type of intense exercise that promotes the exercise capacity of patients with cerebral infarction in a specific rehabilitation process should be identified. However, the formulation of prescribed exercise and the definition of appropriate exercise are currently at a preliminary stage. Since the number of studies concerning the association of movement and memory is limited, further studies for investigating the clinical applicability of such procedures are required.

## Figures and Tables

**Table I:** Comparison of PS amplitude in the hippocampal CA3 area after Y-maze discrimination learning.

Group	After learning 60 times (%)	After mastery (%)
Exercise	78.57±16.32^a^	238.58±18.96
Model	43.12±11.21	235.16±16.46

Data presented are the mean ± standard deviation.

a P<0.05 compared with the model group.

**Table II:** Comparison of the PS incubation period in the hippocampal CA3 area prior to and following Y-maze discrimination learning.

Group	Before (msec)	After (msec)
Exercise	3.08±0.41	3.62±0.18^a^
Model	3.42±0.58	3.09±0.48

Data presented are the mean ± standard deviation.

a P<0.05 compared with the model group.

**Table III:** Comparison of dynamic characteristics acutely isolated from the opening NMDA receptor channel of the hippocampal CA3 neurons in the two groups.

	Opening time		
		
Channel	t1	t2	P-value
Exercise group			
20 pS			
Short opening	0.61±0.33	3.48±2.03	0.108±0.024
Long opening	2.39±0.86	90.9±14.8	0.352±0.109
35 pS			
Short opening	0.89±0.31	3.85±2.46	0.099±0.007
Model group			
20 pS			
Short opening	0.33±0.21^a^	1.89±1.01^a^	0.017±0.009^a^
Long opening	2.03±1.30	30.6±6.98^b^	0.089±0.072^b^
35 pS			
Short opening	0.43±0.24^c^	1.59±0.42^c^	0.036±0.004^c^

Data presented are the mean ± standard deviation.

a P<0.05 compared with 20 pS of short opening in the exercise group;

b P<0.05 compared with 20 pS of long opening in the exercise group;

c P<0.05 compared with 35 pS of short opening in the exercise group.

## References

[1] Lou SJ, Liu JY, Chang H, Chen PJ (2008). Hippocampal neurogenesis and gene expression depend on exercise intensity in juvenile rats. Brain Res.

[2] Gobbo OL, O’mara SM (2005). Exercise, but not environmental enrichment, improves learning after kainic acid-induced hippocampal neurodegeneration in association with an increase in brain-derived neurotrorphic factor. Behav Brain Res.

[3] Albensi BC, Alasti N, Mueller AL Long-term potentiation in the presence of NMDA receptor antagonist arylalkylamine spider toxins. J Neurosci Res.

[4] Chen YZ (1979). Anatomy of rat limbic system.

[5] Qu QM, Cao ZL, Yang JB (2000). Comparison Lpnga method with Koizumi method established rat modals with right middle cerebral artery infarction ischemia. Chinese Journal of Neurology.

[6] Li XM, Li JG, Yang JM (2004). An improved method for acute isolation of neurons from the hippocampus of adult rats suitable for patch-clamping study. Sheng Li Xue Bao.

[7] Watson DJ, Stanton ME (2009). Intrahippocamapal administration of an NMDA-receptor antagonist impairs spatial discrimination reversal learning in weanling rats. Neurobiol Learn Mem.

[8] Yoshihara T, Ichitani Y (2004). Hippocampal N-methyl-D-aspartate receptor-mediated encoding and retrieval processes in spatial working memory: delay-interposed radial maze performance in rats. Neuroscience.

[9] Melik E, Babar E, Ozen E, Ozqunen T (2006). Hypofunction of the dorsal hippocampal NMDA receptors impairs retrieval of memory to partially presented foreground context in a single-trial fear conditioning in rats. Eur Neuropsychopharm.

[10] Berberich S, Jensen V, Hvalby Ø, Seeburg PH, Köhr G (2007). The role of NMDAR subtypes and charge transfer during hippocampal LTP induction. Neuropharmacology.

[11] Gao C, Gill MB, Tronson NC (2010). Hippocampal NMDA receptor subunits differentially regulate fear memory formation and neuronal signal propagation. Hippocampus.

[12] Bartlett TE, Bannister NJ, Collett VJ (2007). Differential roles of NR2A and NR2B-containing NMDA receptors in LTP and LTD in the CA1 region of two-week old rat hippocampus. Neuropharmacology.

[13] Clarke RJ, Johnson JW (2008). Voltage-dependent gating of NR1/2B NMDA receptors. J Physiol.

[14] Lüscher C, Malenka RC (2012). NMDA receptor-dependent long-term potentiation and long-term depression (LTP/LTD). Cold Spring Harb Perspect Biol.

[15] Murphy GG, Glanzman DL (1997). Mediation of classical conditioning in *Aplysia californica* by long-term potentiation of sensorimotor synapses. Science.

[16] Zhang XL, Sullivan JA, Moskal JR, Stanton PK (2008). A NMDA receptor glycine site partial agonist, GLYX-13, simultaneously enhances LTP and reduces LTD at Schaffer collateral-CA1 synapses in hippocampus. Neuropharmacology.

[17] Vaynman S, Ying Z, Gomez-Pinilla1 F (2003). Interplay between brain-derived neurotrophic factor signal transduction modulators in the regulation of the effects of exercise on synaptic-plasticity. Neuroscience.

[18] Dietrich MO, Mantese CE, Porciuncula LO, Ghisleni G, Vinade L, Souza DO, Portela LV (2005). Exercise affects glutamate receptors in postsynaptic densities from cortical mice brain. Brain Res.

[19] Adlard PA, Perreau VM, Engesser-Cesar C, Cotman CW (2004). The timecourse of induction of brain-derived neurotrophic factor mRNA and protein in the rat hippocampus following voluntary exercise. Neurosci Lett.

[20] Liu Y, Wong TP, Aarts M (2007). NMDA receptor subunits have differential roles in mediating excitotoxic neuronal death both in vitro and in vivo. J Neurosci.

[21] Molteni R, Ying Z, Gómez-Pinilla F (2002). Differential effects of acute and chronic exercise on plasticity-related genes in the rat hippocampus revealed by microarray. Eur J Neurosci.

[22] Farmer J, Zhao X, van Praag H, Wodtke K, Gage FH, Christie BR (2004). Effects of voluntary exercise on synaptic plasticity and gene expression in the dentate gyrus of adult male Sprague-Dawley rats in vivo. Neuroscience.

[23] Tsien JZ (2000). Linking Hebb’s coincidence-detection to memory formation. Curr Opin Neurobiol.

[24] Shimizu E, Tang YP, Rampon C, Tsien JZ (2000). NMDA receptor-dependent synaptic reinforcement as a crucial process for memory consolidation. Science.

[25] Baez MV, Oberholzer MV, Cercato MC, Snitcofsky M, Aguirre AI, Jerusalinsky DA (2013). NMDA receptor subunits in the adult rat hippocampus undergo similar changes after 5 minutes in an open field and after LTP induction. PLoS One.

[26] Yu Q, Li XH, Wu SM (2002). The relationship between changes of synaptic interface structure in the contralateral brain area and the ability of learning-memory following motor training in brain ischemic rats. Chin J Phys Med Rehabil.

